# The type VI adenylyl cyclase protects cardiomyocytes from β-adrenergic stress by a PKA/STAT3-dependent pathway

**DOI:** 10.1186/s12929-017-0367-3

**Published:** 2017-09-04

**Authors:** Yu-Shuo Wu, Chien-Chang Chen, Chen-Li Chien, Hsing-Lin Lai, Si-Tse Jiang, Yong-Cyuan Chen, Lin-Ping Lai, Wei-Fan Hsiao, Wen-Pin Chen, Yijuang Chern

**Affiliations:** 1 Graduate Institute of Life Sciences, National Defense Medical Center, Taipei, Taiwan; 2 Institute of Biomedical Sciences, Academia Sinica, Nankang, Taipei, 115 Taiwan; 3 National Laboratory Animal Center, National Applied Research Laboratories, Tainan, Taiwan; 4 Institute of Internal Medicine, College of Medicine, National Taiwan University, Taipei, Taiwan; 5 Institute of Pharmacology, College of Medicine, National Taiwan University, Taipei, Taiwan

**Keywords:** Type V adenylyl cyclase, AC6, cAMP, STAT3, Microdomain

## Abstract

**Background:**

The type VI adenylyl cyclase (AC6) is a main contributor of cAMP production in the heart. The amino acid (aa) sequence of AC6 is highly homologous to that of another major cardiac adenylyl cyclase, AC5, except for its N-terminus (AC6-N, aa 1–86). Activation of AC6, rather than AC5, produces cardioprotective effects against heart failure, while the underlying mechanism remains to be unveiled. Using an AC6-null (AC6^−/−^) mouse and a knockin mouse with AC6-N deletion (AC6 ^ΔN/ΔN^), we aimed to investigate the cardioprotective mechanism of AC6 in the heart.

**Methods:**

Western blot analysis and immunofluorescence staining were performed to determine the intracellular distribution of AC6, AC6-ΔN (a truncated AC6 lacking the first 86 amino acids), and STAT3 activation. Activities of AC6 and AC6-ΔN in the heart were assessed by cAMP assay. Apoptosis of cardiomyocytes were evaluated by the TUNEL assay and a propidium iodine-based survival assay. Fibrosis was examined by collagen staining.

**Results:**

Immunofluorescence staining revealed that cardiac AC6 was mainly anchored on the sarcolemmal membranes, while AC6-ΔN was redistributed to the sarcoplasmic reticulum. AC6^ΔN/ΔN^ and AC6^−/−^ mice had more apoptotic myocytes and cardiac remodeling than WT mice in experimental models of isoproterenol (ISO)-induced myocardial injury. Adult cardiomyocytes isolated from AC6^ΔN/ΔN^ or AC6^−/−^ mice survived poorly after exposure to ISO, which produced no effect on WT cardiomyocytes under the condition tested. Importantly, ISO treatment induced cardiac STAT3 phosphorylation/activation in WT mice, but not in AC6^ΔN/ΔN^ and AC6^−/−^ mice. Pharmacological blockage of PKA-, Src-, or STAT3- pathway markedly reduced the survival of WT myocytes in the presence of ISO, but did not affect those of AC6^ΔN/ΔN^ and AC6^−/−^ myocytes, suggesting an important role of AC6 in mediating cardioprotective action through the activation of PKA-Src-STAT3-signaling.

**Conclusions:**

Collectively, AC6-N controls the anchorage of cardiac AC6 on the sarcolemmal membrane, which enables the coupling of AC6 with the pro-survival PKA-STAT3 pathway. Our findings may facilitate the development of novel therapies for heart failure.

**Electronic supplementary material:**

The online version of this article (doi:10.1186/s12929-017-0367-3) contains supplementary material, which is available to authorized users.

## Background

Type VI adenylyl cyclase (AC6) is one of several transmembrane adenylyl cyclases (ACs) that produce cAMP from ATP during stimulation of Gαs protein-coupled receptors. cAMP is a major cellular messenger that activates the cAMP-dependent protein kinase A (PKA), which phosphorylates a wide variety of cellular proteins [7, 15]. For example, activated PKA directly phosphorylates a number of key calcium-handling proteins that regulate Ca^2+^ stores and cardiac contractility [23], including the ryanodine receptor type 2 (RyR2), phospholamban (PLN), cardiac troponin I (cTn I), and the L-type Ca^2+^ channel (Ca_v_ 1.2) in the heart.

AC6 and the type V adenylyl cyclase (AC5) are the major cardiac AC isoforms and are negatively regulated by free Ca^2+^, Giα, and PKA [2]. A beneficial role of AC6 has been reported in several animal models of heart failure, while AC5 is considered a cause of cardiomyopathy in the aged population [40]. Inhibition of AC5 protects mouse models of heart failure from pressure overload and catecholamine infusion [26, 27]. The possible explanation for the distinct roles of these two ACs in cardiomyocytes included that they may exist at different subcellular locations or couple with an unknown cell survival signaling. Timofeyev and colleagues reported that AC6 is located on the sarcolemmal membrane outside the t-tubule structure, while AC5 is located in the t-tubule, where it interacts with caveolin-3 (CAV3) and phosphodiesterases (PDEs) that mediates the degradation of cAMP [37]. Specific subcellular localization of ACs appears to be important because it may determine the microdomains in which PKA activation occurs and the substrates available for phosphorylation by PKA [1, 10]. For example, the cAMP microdomain is critical for the regulation of potassium currents [5, 6, 36] and L-type Ca^2+^ currents [42] by β-adrenergic stimulation. In addition, Ca^2+^ re-uptake is regulated in the microdomain controlled by the AKAP/PKA/SERCA2a/PLN signalsome [22]. It remains to be elucidated whether the anchorage of AC6 on the sarcolemmal membrane could recruit the signalosome that controls myocyte viability.

We previously reported that the N-terminus of AC6 (AC6-N) is a regulatory domain that modulates its function controlled by Gαi-coupled receptors or protein kinase C [17, 18]. AC6-N also interacts with Snapin, a SNARE-associated protein, and modulates neurite outgrowth via interaction with the Snapin/SNARE complex [43]. The functional role of AC6-N in the heart remains to be elucidated. An earlier clinical trial demonstrated that intracoronary delivering of adenoviruses expressing AC6 to the hearts enhanced heart function [13]. Understanding the mechanism beneath the cardioprotective role of AC6 may further facilitate the development of gene therapy for heart failure. In the present study, we employed an AC6-null (AC6^−/−^) mouse and a knockin mouse (AC6^ΔN/ΔN^) expressing a AC6 variant that lacks a critical domain of the N-terminus (aa 2–86, designated AC6-ΔN) to characterize the mechanism underlying the protective role of AC6 after exposure to excessive β-adrenergic stimulation.

## Methods

### Animals

The mouse AC6 gene was isolated from an RPCI-23 female (C57BL/6 J) mouse BAC library (Children’s Hospital Oakland Research Institute, Oakland, CA, USA; clone no. 34P24). The target vector was created using recombineering technology as previously described [21]. In the AC6^ΔN/ΔN^ target vector, a DNA fragment encoding amino acids (aa) 2–86 was replaced in frame with a flag sequence, and a neo cassette was inserted into intron 2 of AC6. AC6^ΔN/ΔN^ mice were generated by the transgenic core facility (TCF) of Academia Sinica (Taipei, Taiwan). The chimeras were crossed with 129S6 mice to maintain the germ line. Genotyping using DNA extracted from tails was carried out by standard PCR (primers are marked with red arrowheads in Additional file 1: Figure S1a; the sequences are: P1: 5′-AACGCAATGGGCAGAAGCGC-3′, P2: 5′-GATTACAAGGATGACGACGATAAG-3′; P3: 5′-ACCTGCACAAGCCGGT- GCC-3′). Expressions of full-length AC6 (WT) and AC6^ΔN^ transcripts in the heart were examined by RT-PCR using primers (P4 and P5, respectively) located in the exon 1 and the exon 3. Compared with that amplified from WT transcripts, the fragment amplified from AC6^ΔN^ transcripts was smaller (Additional file 1: Figure S1c). Sequence of this fragment indicated the successful deletion of exon 2 and the insertion of a flag-tag sequence (Additional file 1: Figure S1d).

Mice were housed in the Institute of Biomedical Science Animal Core Facility (Taipei, Taiwan) at a 12-h light/dark cycle. All animal experiments were performed under protocols, which conform the NIH guidelines, approved by the Academia Sinica Institutional Animal Care and Utilization committee, Taipei, Taiwan. No detectable difference in the body weight of AC6^ΔN/ΔN^ mice and their littermate controls (WT) was detected. All experiments were conducted using mice at 2–5 months of age.

### cAMP measurement

cAMP content was measured as previously reported [8] with modification. Briefly, mouse hearts were dissected and homogenized with a glass homogenizer in ice-cold lysis buffer (20 mM Tris-HCl pH 8.0, 0.5% vol/vol Nonidet P-40, 150 mM NaCl, 1 mM EDTA, 1X PhosSTOP phosphatase inhibitor cocktail, and 10 μM PMSF). After the removal of nuclei and debris by centrifugation at 1300 x *g* for 10 min, the protein concentrations were assayed using the Bio-Rad Protein Assay Dye Concentrate (Bio-Rad, Hercules, CA, USA). cAMP was extracted by adding 300 μl of 0.1 N HCl to each tube with gentle mixing for 10 min on ice. cAMP content was assayed by a ^125^I–cAMP assay kit (PerkinElmer Life Sciences, Shelton, CT, USA) according to the manufacturer’s instructions.

### SDS-polyacrylamide gel electrophoresis (PAGE) and western blot analysis

Total protein lysates were harvested from mouse hearts and subjected to SDS-PAGE and western blot analyses as described previously [8] using the following primary antibodies: AC6D (1: 2000, (18)), anti-flag (1:1000, Sigma-Aldrich, St. Louis, MO, USA), anti-AC5 (1:500, [41]); anti-Ca_v_ 1.2 (1:2000, GeneTex, Irvine, CA, USA); anti-SERCA2a (1:5000, Abcam, Cambridge, UK); anti-RyR2 (1:1000, Alomone Labs, Jerusalem, Israel); anti-p 705-STAT3(1:1000, Biolegend, San Diego, CA, USA) anti-STAT3 (1:1000, Cell signaling, Danvers, MA, USA).

### Adult cardiomyocyte isolation

Mice were anesthetized with urethane (1 g/kg, intraperitoneal injection) and heparin (200 IU). Mouse hearts were quickly excised and incubated in a Ca^2+^-free Tyrode solution (135 mM NaCl, 4 mM KCl, 1 mM MgCl_2_, 10 mM HEPES, and 0.33 mM NaH_2_PO_4_). The ascending aorta was cannulated with a 26G–PE tube. The heart was mounted on a Langendorff apparatus for retrograde perfusion with a pre-warmed (37 °C) Ca^2+^-free Tyrode-HEPES buffer at 3 ml/min for 3 min to wash out blood, followed by a Ca^2+^-free Tyrode-HEPES buffer containing BDM (10 mM) and an enzyme cocktail (collagenase D 0.2 mg/ml, collagenase B 0.3 mg/ml, and Proteinase IX V 0.04 mg/ml) for another 7 min. The heart was then cut into small pieces and further dissociated by pipetting in Ca^2+^ free Tyrode-HEPES buffer containing BDM (10 mM) and BSA (1%). The cell mixtures were passed through a 250-μm mesh filter and allowed to sit at room temperature (RT) for 30 min. The isolated cardiomyocytes were gradually recovered by passage through a Ca^2+^ gradient (0.09, 0.36, 0.9, and 1.8 mM; 5 min per concentration) in Tyrode-HEPES buffer. Cells were stored in 1.8 mM Tyrode-HEPES buffer at RT to recover sarcoplasmic reticulum (SR) Ca^2+^ stores before use.

### Cardiomyocyte culture and survival assay

Freshly isolated adult cardiomyocytes (3 × 10^4^ cells / 3- cm diameter well) were suspended in plating medium (22.7 mM NaHCl, 2 mM L-glutamine, 10 mM blebbistatin, 5% FCS, and 100 U/ml penicillin in MEM medium) and seeded on laminin (Invitrogen, Carlsbad, CA, USA)-coated plates. Cells were treated with or without isoproterenol (10 μM) in the absence or presence of cucurbitacin I (500 nM; 30 min pretreatment) or KT-5720 (1 μM; 30 min pretreatment) as indicated on day 1 and cultured for 24 h. On day 2, the living cells were loaded with Hochest-33,342 dye (Hoechst, 5 μg/ml, Thermo, Rockford, IL,USA) at 37 °C for 90 min, and dead cells were labeled with propidium iodine (PI, 10 μg/ml, Sigma, Saint Louis, MO, USA) for 30 s at RT. After extensive washing, cells were fixed with 4% paraformaldehyde for imaging using a microscope equipped with a digital camera (Axiovert 200 M, Carl Zeiss, Germany). The Hoechst-positive and PI-negative cells with uncondensed chromatin were defined as surviving cells. ImageJ software (Bethesda, MD, USA) was used to analyze the percentage of surviving cells under the experimental conditions tested. At least 30 cells were scored in each experiment. For the STAT3 activation experiment, cells were treated with or without isoproterenol (10 μM, 15 min) in the absence or presence of KT-5720 (1 μM, 15 min; 30 min pretreatment) or Src inhibitor-1 (1 μM, 15 min; 5 min pretreatment) as indicated on day 1. After extensive washes, cells were fixed with 4% paraformaldehyde, subjected to immunocytochemistry using the STAT3 antibody (Cell Signaling) as described below.

### Immunocytochemistry

Isolated cardiomyocytes were fixed with 4% paraformaldehyde and permeabilized with 0.2% Triton X-100. Endogenous hydroperoxidase activity was blocked using 1.5% H_2_O_2_. After blocking with 3% bovine serum albumin plus donkey anti-mouse IgG for 1 h, myocytes were incubated with the following primary antibodies: N20 (1:50, Santa Cruz Biotechnology), anti-flag (1:100, Sigma-Aldrich), anti-Src (1:100, Cell Signaling), anti-α-actinin (1:200, Abcam), anti-Ca_v_ 1.2 (1:100, GeneTex), or anti-SERCA2a (1:100, Abcam) at 4 °C over night. After extensive washes in phosphate buffered saline (PBS; 137 mM NaCl, 2.7 mM KCl, 10 mM Na_2_HPO_4_, and 1.8 mM KH_2_PO_4_), cells were incubated with a fluorescein-conjugated secondary antibody. To amplify the signal of endogenous AC6, cardiomyocytes labeled with the anti-AC6 antibody (N20, Santa Cruz Biotechnology) were incubated with a horseradish peroxidase-conjugated secondary antibody, and the signal was enhanced using a Tyramide Signal Amplification kit (PerkinElmer, MA, USA). Nuclei were stained with DAPI. The fluorescent images were acquired via a laser-scanning confocal microscope (LSM 510 meta, Carl Zeiss, Germany).

### Collagen staining for fibrosis detection

Mice were anesthetized using urethane (Sigma-Aldrich, 1 g/kg, intraperitoneal injection) and perfused with 4% (*w*/*v*) paraformaldehyde. Their hearts were excised and post-fixed in 4% (*w*/*v*) paraformaldehyde, followed by a paraffin-embedding procedure using a standard protocol. Transverse cardiac sections of 5 μm were cut and subjected to Sirius red staining to identify the collagen-containing region. Six sections from each heart were analyzed and images were taken using a microscope (BX-51: Olympus, Center Valley, PA, USA). The red area was determined using Image-Pro plus (Media Cybernetics, Rockville, MD, USA) and normalized to the total cardiac area of the heart sections.

### TUNEL assay

Mouse hearts were dissected and the cardiac sections were prepared as described above. TUNEL assay was carried out using a DeadEnd Fluorometric TUNEL system (Promega, Madison, WI, USA) according to the manufacturer’s instructions.

### Statistical analysis

All data are expressed as the mean ± SEM. Unless stated otherwise, statistical significance was evaluated using an unpaired Student’s *t*-test or a one-way analysis of variance (ANOVA) followed by the Fisher LSD post-hoc test using Sigmaplot software (Version 3.1; Systat Software Inc). *P* values <0.05 were considered statistically significant.

## Results

### Mice lacking AC6 or the N-terminal domain (aa 2–86) of AC6 developed severe cardiac remodeling after exposure to β-adrenergic stimulation

**Fig. 1:**
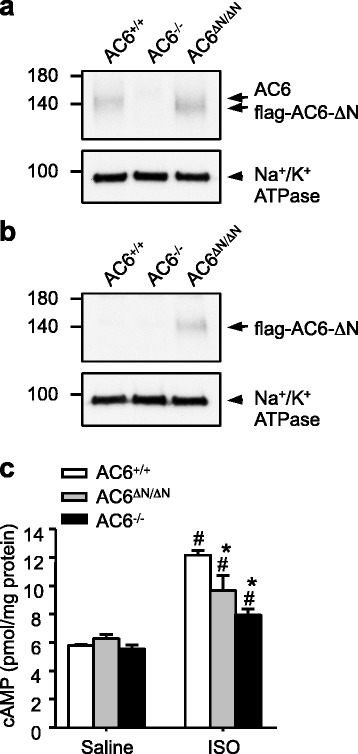
Compared with WT mice (AC6^+/+^), hearts of AC6^ΔN/ΔN^ and AC6^−/−^ mice had lower cAMP levels evoked by βAR-stimulation. Total lysates were prepared from the hearts of the indicated mice (2–5 months old). **a, b** Expression of AC6 and flag-AC6-ΔN was detected by Western blot analysis using anti-AC6 (AC6D, **a**) and anti-flag (**b**) antibodies, respectively. Na^+^/K^+^ − ATPase was used as a loading control. **c** The cAMP level in total lysate evoked by ISO (10 mg/kg, i.p. injection 15 min; AC6^+/+^, *N* = 7; AC6 ^ΔN/ΔN^, *N* = 4; AC6^−/−^, *N* = 3) was measured and normalized to the total amount of protein. The data are presented as the mean ± SEM.^*^ *P* < 0.05 vs. AC6^+/+^. ^#^ *P* < 0.05 vs. the saline group

We had previously created an AC6^−/−^ mouse to study the physiological function of AC6 [9]. Our earliest studies suggest that the N terminal domain (aa 2–86, designated AC6-N) of AC6 is the most variable region of AC6 and functions as a regulatory domain [18]. In the present study, we further designed and generated a mouse model (designated AC6^ΔN/ΔN^) that expressed an AC6 mutant (AC6-ΔN) lacking aa 2–86 (Additional file 1: Figure S1). A flag-tag was inserted at N-terminus of AC6-ΔN to enable the detection of mutant protein. The expression levels of AC6 variants in the heart were analyzed using an anti-AC6 antibody (AC6D, [19]) that recognized the C-terminus of AC6. As shown in Fig. 1a, an immunopositive band of 150 kDa was observed in WT mice (AC6^+/+^), while a slightly smaller band was observed in AC6^ΔN/ΔN^ mice. No immunopositive band corresponding to AC6 was found in the heart of AC6^−/−^ mice [9]. Of note, the immunopositive bands of AC6 variants appear slightly fuzzy in western blot analysis because AC6 is glycosylated at Asn^805^/Asn^890^ [44]. Using an anti-flag antibody, only one band corresponding to flag-AC6-ΔN was detected in AC6^ΔN/ΔN^ mice (Fig. 1b). No compensatory change in the expression of AC5 protein was detected in the hearts of AC6^ΔN/ΔN^ mice (Additional file 1: Figure S1e). In most animals tested, the expression level of AC6-ΔN is slightly higher than that of AC6, suggesting that the N terminal domain of AC6 may be important for its protein stability.

When compared to the heart of wild-type mice, no change in the basal cAMP level in AC6^ΔN/ΔN^ and AC6^−/−^ mice was observed. Importantly, the cAMP level evoked by isoproterenol (ISO, an agonist of β-ARs; 10 mg/kg, intraperitoneal injection (i.p.), 15 min) in the hearts of AC6^ΔN/ΔN^ and AC6^−/−^ mice were significantly lower than that of wild-type mice (Fig. 1c), suggesting that AC6 functions downstream of β-ARs. In addition, the β-ARs-AC6-cAMP-PKA signaling in the hearts of AC6^ΔN/ΔN^ and AC6^−/−^ mice appeared to be altered.

**Fig. 2:**
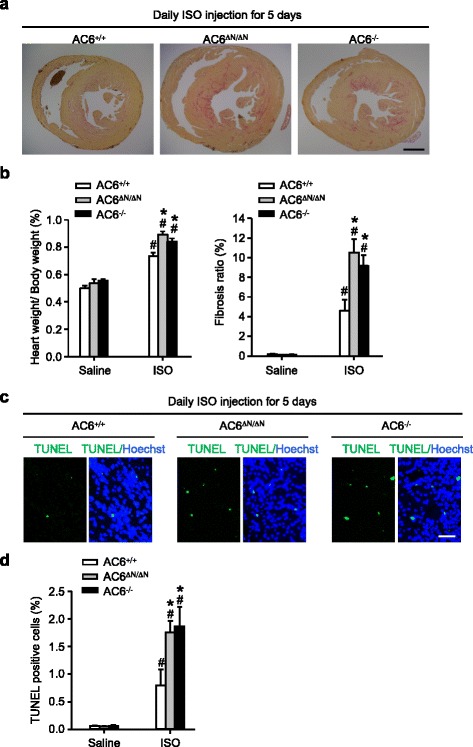
Severer cardiac remodeling in hearts of AC6^ΔN/ΔN^ and AC6^−/−^ mice. Mice were daily injected with ISO (10 mg/kg/day) or saline for 5 days. Hearts were carefully removed, weighted, and used to prepare heart sections, which were then subjected to collagen stain and TUNEL assay. **a** Representative pictures of collagen stain. Scale bar, 1 mm. **b** Quantitation of heart weight, and fibrosis ratio. AC6^+/+^/saline, *N* = 4; AC6^ΔN/ΔN^/saline, *N* = 3; AC6^−/−^/saline, *N* = 3; AC6^+/+^/ISO, *N* = 6; AC6^ΔN/ΔN^/ISO, *N* = 4; AC6^−/−^/ISO, *N* = 4. ^*^ *P* < 0.05, vs. AC6^+/+^. ^#^ *P* < 0.05, vs. the saline group. **c** Representative pictures of TUNEL assay. Scale bar, 50 μm. **d** Quantitation of TUNEL-positive cells. *N* = 3 in each group. ^*^ *P* < 0.05, vs. AC6^+/+^. ^#^ *P* < 0.05, vs. the saline group

To determine whether AC6^ΔN/ΔN^ and AC6^−/−^ cardiomyocytes were more sensitive than wild-type cardiomyocytes to the stress response evoked by β-stimulation, mice were subjected to a single dose of ISO (10 mg/kg, i.p. injection), allowed to rest for 7 days, and harvested for further analyses. As shown in Additional file 1: Figure S2, acute ISO treatment caused no detectable cardiac hypertrophy and a mild fibrosis in WT mice. In contrast, this ISO treatment greatly enhanced the extent of fibrosis, apoptosis, and caused cardiac hypertrophy in AC6^ΔN/ΔN^ and AC6^−/−^ mice (Additional file 1: Figure S2a-c). We next evaluated the cardiac remodeling of these mice using a more stressful condition. Mice were subjected to daily ISO injection (10 mg/kg, i.p. injection) for consecutive five days. On day 5, cardiac hypertrophy, fibrosis, and apoptosis in AC6^ΔN/ΔN^ and AC6^−/−^ mice were much more severe than WT mice (Fig. 2). Interestingly, removal of AC6 entirely (AC6^−/−^) behaved similarly to AC6^ΔN/ΔN^ mice, and exhibited severe cardiac remodeling in response to ISO treatment (Fig. 2a). Collectively, the loss of AC6 or AC6-N lowered the viability of cardiomyocytes and increased cardiac remodeling in response to β-AR stimulation.

### AC6-N determined the subcellular localization of AC6 in adult cardiomyocytes

**Fig. 3:**
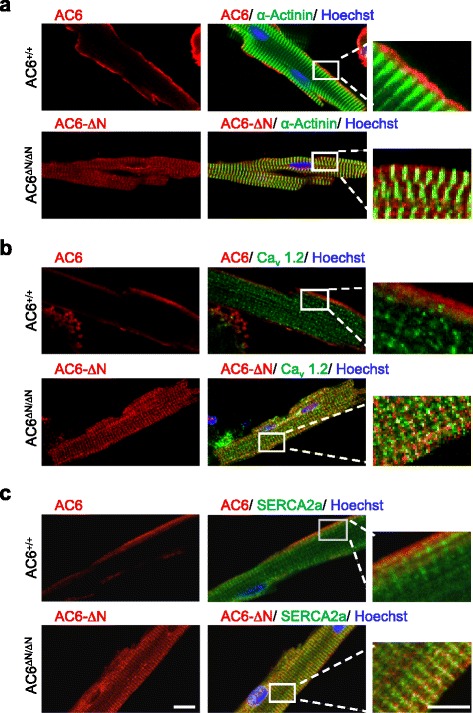
Cellular localization of AC6 and flag- AC6-ΔN in cardiomyocytes. Localization of AC6 (red) or flag- AC6-ΔN (red), and α-actinin (**a**, green), Ca_v_1.2 (**b**, green), and SERCA2a (**c**, green) in adult cardiomyocytes was assessed by immunofluorescence staining. Scale bar, 10 μm. The rightmost panels show the enlarged, merged images of fields in white rectangles. Scale bar, 5 μm

We have previously demonstrated that AC6-N is a regulatory domain of AC6 and does not directly contribute to its catalytic activity [18]. To assess whether AC6-N played an important role in the subcellular localization of AC6, adult cardiomyocytes were isolated and subjected to immunofluorescence staining. The full-length AC6, which was detected using an anti-AC6 antibody (N20), was located at the sarcolemmal membrane (Fig. 3a). Conversely, the flag-AC6-ΔN protein detected using an anti-flag antibody was located intracellularly (Fig. 3a). Co-immunostaining of flag-AC6-ΔN and the L-type calcium channel (a marker of the t tubule) or the sarcoplasmic reticulum Ca^2+^-ATPase (SERCA2a, a marker of SR) further demonstrated that flag-AC6-ΔN was co-localized with SERCA2a in the SR but not in the t-tubules (Fig. 3b-c, Additional file 1: Figure S3-S4). The change of cellular distribution of AC6-ΔN was coincident with the reduced cAMP after ISO stimulation (Fig. 1b).

### AC6 mediated Src-dependent STAT3 activation on the sarcolemma and protected cardiomyocytes against cardiac stress through a PKA/STAT3-dependent pathway

**Fig. 4:**
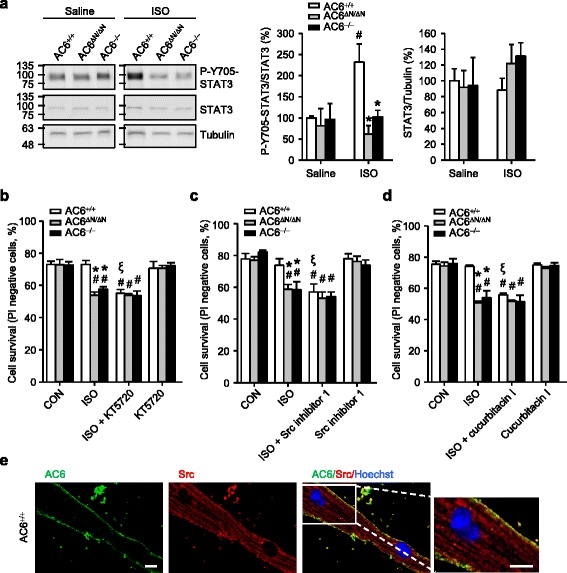
AC6-N regulates the PKA and Src-mediated STAT3 phosphorylation and cardiomyocyte survival. **a** The indicated mice were subjected to an acute ISO treatment (10 mg/kg, i.p.). Fifteen minutes after the injection, the heart was removed carefully to prepare total lysates and analyzed using Western blot analyses. The levels of phosphorylated STAT3 (P-Y705-STAT3), STAT3, and Tubulin were detected using the indicated antibody. Tubulin was used as a loading control. The phosphorylation level of STAT3 was normalized to the amount of the total STAT3, while the amount of the total STAT3 was normalized to the loading control (α-tubulin, *N* = 4 per group). **b**, **c**, **d** Adult cardiomyocytes were treated with isoproterenol (ISO, 10 μM) in the absence or presence of a PKA inhibitor (KT-5720, 1 μM, 30 min pretreatment; **b**), a Src inhibitor (Src inhibitor 1, 1 μM, 5 min pretreatment; **c**), or a STAT3 inhibitor (cucurbitacin I, 500 nM, 30 min pretreatment; **d**) for 24 h. The hoechst-positive and propidium iodine (PI)-negative with uncondensed chromatin cells were defined as surviving cells. The data are expressed as the mean ± SEM (*N* = 3, **b**; *N* = 4, **c**; *N* = 3, **d**). ^*^ *P* < 0.05 vs. AC6^+/+^ in the indicated condition. ^#^ *P* < 0.05 vs. the basal level in each group. ^ξ^ *P* < 0.05 vs. the ISO-treated cells in each group. **e** Adult cardiomyocytes were immunostained for AC6 (green) and Src (red). Scale bar, 10 μm. The rightmost panels show the enlarged, merged images of fields in white rectangles. Scale bar, 10 μm

An earlier study suggests that high concentrations of catecholamine activates the Src/STAT3 pathway to avoid apoptosis [47]. To assess whether STAT3 is involved in the function of AC6, we examined the effect of ISO treatment (10 mg/kg, i.p. injection, 15 min) on the levels of STAT3 phosphorylation in the heart [47]. As shown in Fig. 4a, the levels of ISO- induced STAT3 phosphorylation in the hearts of AC6^ΔN/ΔN^ and AC6^−/−^ mice were significantly lower than that of WT mice, indicating that AC6 on the sarcolemma might mediate the STAT3 pathway. In order to clarify whether the AC6-mediated STAT3 activation contributed to cardiomyocyte survival under catecholamine stress, we next evaluated whether adult cardiomyocytes harvested from WT, AC6^ΔN/ΔN^, or AC6^−/−^ mice responded differently to β-AR stimulation for cardiac apoptosis

Adult cardiomyocytes were isolated, cultured for 24 h, treated with ISO (10 μM) in the absence or presence of a PKA inhibitor (KT-5720, 1 μM, 30 min pretreatment; Fig. 4b), a Src inhibitor (Src inhibitor 1, 1 μM, 5 min pretreatment; Fig. 4c), and a STAT3 inhibitor (cucurbitacin I, 500 nM, 30 min pretreatment; Fig. 4d) for 24 h. The living cells were identified as hoechst-positive and propidium iodine (PI)-negative, while the dead cells were hoechst-positive and PI-positive. No significant difference in the survival of adult cardiomyocytes isolated from WT, AC6^ΔN/ΔN^, or AC6^−/−^ mice was detected at the basal condition (Fig. 4b–d). Nonetheless, treatment with ISO significantly reduced the survival of adult cardiomyocytes of AC6^ΔN/ΔN^ and AC6^−/−^ mice, but not those of wild-type mice. Importantly, treatment with KT5720, Src inhibitor 1, or cucurbitacin I suppressed the survival of WT myocytes to a level comparable to those of the ISO-treated AC6^ΔN/ΔN^ and AC6^−/−^ cardiomyocytes (Fig. 4b–d, respectively). No further suppressing effect of KT-5720, Src inhibitor 1 and cucurbitacin I on the survival of AC6^ΔN/ΔN^ and AC6^−/−^ myocytes was observed (Fig. 4bd-). KT-5720, Src inhibitor 1, or cucurbitacin I alone did not produce any effect on the survival of all myocytes tested. Collectively, these findings suggest that the β-AR- induced PKA- and STAT3- dependent survival signal are absent in cardiomyocytes of both AC6^ΔN/ΔN^ and AC6^−/−^ mice. Double immunofluorescence assay revealed that AC6 was colocalized with Src, an upstream regulator of STAT3 [4], on the sarcolemma (Fig. 4e), indicating that AC6 might mediate the activation of STAT3 via interacting with Src. No clear co-localization of AC6-N and Src was observed (Additional file 1: Fig. S5).

**Fig. 5:**
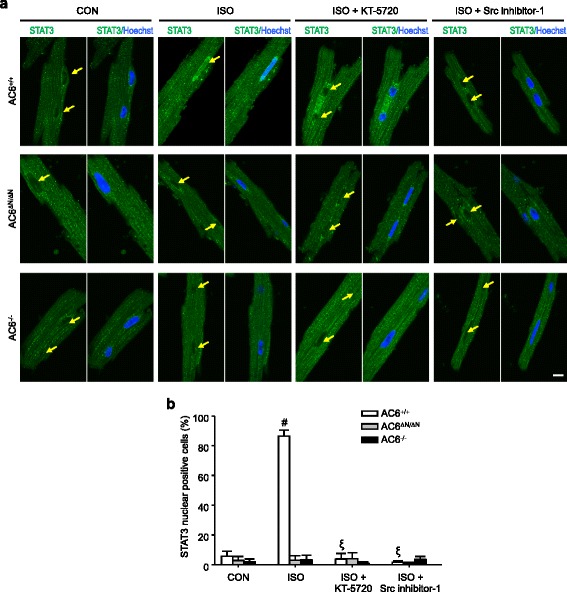
The AC6-N regulates the activation of STAT3 in a PKA- dependent manner. **a** Adult cardiomyocytes were pretreated with or without a PKA inhibitor (KT-5720, 1 μM, 30 min pretreatment) or an Src inhibitor (Src inhibitor 1, 1 μM, 5 min pretreatment) as indicated, followed by a 15- min incubation with isoproterenol (ISO, 10 μM). The scale bar, 10 μm. **b** The ratios of nuclear STAT3- positive cells in each condition were quantified. Three to four independent experiments were conducted in each condition. At least 30 cardiomyocytes in each condition/experiment were scored. The data are expressed as the mean ± SEM. ^*^ *P* < 0.05 vs. AC6^+/+^ in each group. ^#^ *P* < 0.05 vs. the basal level in each group. ^ξ^ *P* < 0.05 vs. the ISO-treated cells in each group

To further clarify the role of AC6 in mediating the activation of STAT3 by β-AR stimulation, we next examined whether the ISO-evoked STAT3 activation requires AC6, PKA, or Src. As shown in Fig. 5, treatment with ISO (10 μM, 15 min) induced the accumulation of STAT3 in the nuclei of cultured WT myocytes, but not in those of AC6^ΔN/ΔN^ and AC6^−/−^ mice. Inhibition of PKA (KT-5720, 1 μM, 15 min) or suppression of Src (Src inhibitor-1, 1 μM, 15 min) prevented the activation of STAT3, as assessed by the nuclear accumulation of STAT3, of WT cardiomyocytes. KT-5720 or Src inhibitor-1 did not alter STAT3 activation in AC6^ΔN/ΔN^ or AC6^−/−^ myocytes, supporting that sarcolemmal AC6 is located upstream of the prosurvival PKA/Src/STAT3 pathway.

Collectively, our findings suggest that AC6-N anchors AC6 on the sarcolemma, where AC6 might mediate the activation of the PKA/Src/STAT3 pathway and protect cardiomyocytes from apoptosis triggered by cardiac stress.

## Discussion

The PKA-mediated β-AR signaling is one of the key pathways that regulates multiple cardiac functions, including positive inotropic, lusitropic and chronotropic effects [45]. The role of ACs in mediating cAMP production and PKA activation in response to β-AR stimulation is thus critical in the heart [31]. Both AC6 and AC5 are the major AC isotypes in the heart. Though AC6 and AC5 share almost identical amino acid sequences except for their N-terminal domains [18], the N-termini of AC6 and AC5 appear to be the key components in controlling their distinct subcellular localizations, and in turn determine their functions in regulating cardiac contractile performance and/or stress response. A previous report revealed that AC6 is located on the sarcolemmal membrane except for at t-tubules, and that AC5 exists in t-tubules and forms signalosomes with βAR and PDE via an interaction with caveolin 3 [37]. The deletion of AC6 results in abnormal Ca^2+^ handling, while ablation of AC5 protects animals from heart disease [27, 33]. Consistent with a detrimental role for AC5, its overexpression activates the cAMP/PKA activity in t-tubules and phosphorylates the nearby L-type calcium channel in t-tubules, causing arrhythmia [48]. Conversely, AC6 is located on the sarcolemmal membrane out of t-tubules. The present study demonstrated that the regulatory N-terminal domain of AC6 (AC6-N) determines its sarcolemmal anchorage to concomitantly mediate two functional responses of both positive inotropism and cell survival in response to β-adrenergic stimulation. Loss of AC6-N could lead to the absence of AC6 in the sarcolemmal membrane and caused more cell death under continuous β-AR stimulation (Figs. 2, 3, 4 and 5). In addition to the critical localization of AC6 in the sarcolemmal membrane, the most variable N terminal domain (aa 2–86) of AC6 might also harbor important domain(s) that mediate the activation of Src/STAT3 signaling. The functional importance of AC6-N warrants further investigation. Several earlier studies reported that overexpression of AC6 in the heart could protect animals from heart failure induced by a pace-maker, ISO, or aging via increasing the efficiency of βAR-AC6 coupling, thereby activating the downstream PKA-signaling cascade that produces a significantly positive inotropic effect to ameliorate the function of a failing heart and lower sympathetic tone via a baroreflex [12, 20, 35]. Our study highlighted the important role of AC6-N in concomitantly elevating the cardiac stress tolerance through coupling a PKA-Src-STAT3-dependent cascade against cardiac stress. These findings also unveiled a novel druggable target to increase cardiomyocyte viability beyond producing the positive inotropic effect on heart failure.

Using the knock-in mouse model with the N-terminal truncation of AC6 (aa 2–86 deletion, AC6^ΔN/ΔN^), we found that the consecutive administration of ISO for 5 days caused more cardiomyocyte death and more severe cardiac remodeling in both AC6^ΔN/ΔN^ and AC6^−/−^ mice as compared to those in WT group (AC6^+/+^) (Fig. 2a). Immunofluorescence revealed that AC6-ΔN was redistributed from the sarcolemma onto SR (Fig. 3). We also assessed the calcium-handling function in WT and AC6^ΔN/ΔN^ cardiomyocytes. The current-voltage relation curves of L-type calcium channels showed no significant difference between adult WT and AC6^ΔN/ΔN^ cardiomyocytes (Additional file 1: Figure S6a-b). No change in the expression level of calcium-handling proteins such as RyR, L-type calcium channel, and SERCA2 was detected in the hearts of AC6^ΔN/ΔN^ mice either (Additional file 1: Figure S6c-e). Since there is no significant alteration in the current density of the L-type calcium channel in AC6^ΔN/ΔN^ cardiomyocytes (Additional file 1: Figure S6a, 4b), the redistribution of AC6-ΔN out of the sarcolemma did not alter the functional coupling between the L-type calcium channel and the signalosome of βAR-AC5 in t-tubules. Another interesting observation is that, despite the basal cAMP content in the heart of AC6^ΔN^ mice was unaffected, echocardiographic measurements showed that the basal cardiac function of AC6^ΔN/ΔN^ mice was slightly less than wild-type mice (Additional file 1: Table S1). It is also interesting to note that AC5 coupled to β1AR promotes cardiac remodeling, while AC6 coupled to β2AR activates cell survival pathways [39]. Whether β2-agonists [16, 24, 25, 29] or β-blockers with intrinsic sympathomimetic activity evoke the AC6-STAT3 cascade in the heart remains to be further elucidated [3, 14].

Since AC6 is absent from the sarcolemmal membrane in the hearts of AC6^ΔN/ΔN^ mice, we hypothesized that β-AR stimulation evoked the cAMP/PKA signaling from other ACs in these mice. Consistently, the β-adrenergic stimulation evoked less cAMP/PKA signaling in the heart of AC6^ΔN/ΔN^ mice when compared with that of WT (Fig. 1c). Removal of AC6 entirely (AC6^−/−^) also reduced the production of cAMP during β-AR stimulation (Fig. 1c). This is of great importance because treatment with ISO for 24 h induced more cell death in AC6^ΔN/ΔN^ and AC6^−/−^ myocytes than in WT myocytes. The ISO-induced cell death in WT myocyte could be aggravated in the presence of the inhibitor of PKA, Src, or STAT3, consolidating the involvement of these molecules in the β-AR stimulation-evoked cardiac death (Fig. 4). We are particularly interested in STAT3 because it has been implicated in a cardioprotective effect on cardiomyocyte during β-AR stress [47]. The lower STAT3 phosphorylation/activation during β-AR stimulation in AC6^ΔN/ΔN^ and AC6^−/−^ mice indicated that AC6 on the sarcolemma might play a role in mediating STAT3 activation in the heart. Because Src is required for the β-AR- mediated activation of STAT3 [28], and since that Src can be phosphorylated and activated by PKA [32], we hypothesized that Src may play a critical role in coupling the βAR-AC6-PKA and the STAT3 pathways, possibly via docking the signalosome on AKAP79 [46]. Our data support that Src is colocalized with AC6 and that it is required for the AC6-dependent STAT3 activation during β-AR stimulation (Figs. 4e and 5b).

AC6 has been an important therapy target for heart failure in preclinical and clinical trials [11, 30, 34]. The initial clinical study of intracoronary delivery of AC6 to the hearts of patients with heart failure resulted in marked beneficial effect [13]. Results of the present study identified an important cell survival signaling mediated by AC6 under β-adrenergic stimulation, which provides an explanation for the beneficial effect of AC6 overexpression in failing hearts. The N-terminus of AC6 is likely to anchor AC6 on the sarcolemmal membrane and activates the Src/STAT3 signaling to prevent death of myocytes induced by β-adrenergic stress in heart failure patients who usually have excessive sympathetic reflex [38].

## Conclusions

**Fig. 6:**
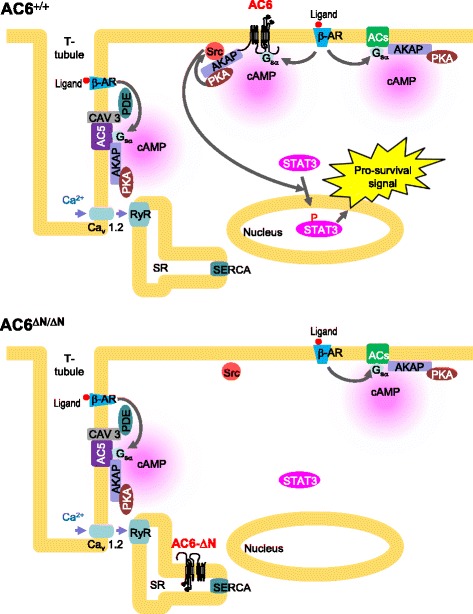
Schematic diagram of AC6 and AC6-ΔN signaling. The AC6 signaling pathway in a WT cardiomyocyte (AC6^+/+^) is shown in the upper panel. AC6 normally anchors on the sarcolemmal membrane and interacts with AKAP and PKA. The lower panel shows the signaling pathway in a cardiomyocyte (AC6^ΔN/ΔN^) that expresses AC6-ΔN (an AC6 variant that lacks aa 2–86), but not the full-length AC6. Our data suggest that AC6-ΔN re-distributes from the sarcolemmal membrane to the sarcoplasmic reticulum (SR), indicating a role of AC6-N in the anchorage of AC6 on the sarcolemmal membrane. Our data also suggest that AC6 locates on the sarcolemmal membrane, and allows the PKA/Src/STAT3 pathway to be activated by β adrenergic receptors (β-ARs). While the AC6-ΔN variant is redistributed to SR, the AC6/PKA/Src/STAT3 pathway is unable to be activated during cardiac stress, and consequently results in poor survival of cardiac myocytes

The present study clarified a novel role of the sarcolemmal AC6 in supporting cardiomyocyte survival via activating the AC6-PKA-Src-STAT3 signaling (Fig. 6). Our data suggest that AC6-N determines the membrane anchorage and the consequent transduction of a survival signaling. Results of the present study may facilitate the development of novel drugs for post-injured heart or heart failure.

## Additional file

Additional file 1: Supplementary material.ᅟ(PDF 7602 kb)

## Abbreviations

AC5: type V adenylyl cyclase
AC6: type VI adenylyl cyclase
AC6-N: N-terminus of AC6
AC6^ΔN^: N-terminus deleted AC6
AKAP: A-kinase anchor protein
CAV3: caveolin-3
cTn I: cardiac troponin I
EDD: end-diastolic diameter
EF: ejection fraction
ESD: end-systolic diameter
FK: forskolin
FS: fraction shorting
HR: heart rate
ISO: isoproterenol
IVS: interventricular septum
PDE: phosphodiesterases
PKA: protein kinase A
PLN: phospholamban
PW: posterior wall
RyR2: ryanodine receptor type 2
SERCA2a: sarcoplasmic reticulum Ca^2+^ ATPase type 2a
SR: sarcoplasmic reticulum
β-AR: β-adrenergic receptor
Src: Src kinase
STAT3: Signal transducer and activator of transcription 3
