# Supplementary material for: The type VI adenylyl cyclase protects cardiomyocytes from β-adrenergic stress by a PKA/STAT3-dependent pathway
Source: J Biomed Sci. 2017 Sep 4;24:68. doi: 10.1186/s12929-017-0367-3 (PMC5584049; doi:10.1186/s12929-017-0367-3)
Supplement: Supplementary file 1 — Supplementary material.ᅟ(PDF 7602 kb) [file 12929_2017_367_MOESM1_ESM.pdf]

SUPPLEMENTARY MATERIAL

Full title: **The type VI adenylyl cyclase protects cardiomyocytes from beta-adrenergic stress by a PKA/STAT3-dependent pathway**

Authors: Yu-Shuo Wu[1,2], Chien-Chang Chen[1,2], Chen-Li Chien[1,2], Hsing-Lin Lai[2], Si-Tse Jiang[3], Yong-Cyuan Chen[2], Lin-Ping Lai[4], Wei-Fan Hsiao[5], Wen-Pin Chen[5,*] and Yijuang Chern[1,2,*]
(*, *co-corresponding authors.*)

Affiliations: [1] Graduate Institute of Life Sciences, National Defense Medical Center, Taipei, Taiwan. [2]Institute of Biomedical Sciences, Academia Sinica, Taipei, Taiwan. [3] National Laboratory Animal Center, National Applied Research Laboratories, Tainan, Taiwan. [4] Institute of Internal Medicine, Colleague of Medicine, National Taiwan University, Taipei, Taiwan. [5] Institute of Pharmacology, Colleague of Medicine, National Taiwan University, Taipei, Taiwan.

Supplemental Methods

RNA and reverse-transcription (RT) polymerase chain reaction (PCR)

Total RNA was extract from the heart of WT ($AC6^{+/+}$) and $AC6^{\Delta N/\Delta N}$ mice using TRIZOL Reagent (Invitrogen, Carlsbad, CA, USA). Complementary (c)DNA was reverse-transcribed from RNA by Random hexamer priming (Promega, WI, USA) and SuperScript III reverse transcriptase (Invitrogen). Reverse-transcription (RT) PCR for messenger RNA of $AC6^{+/+}$ or $AC6^{\Delta N/\Delta N}$ from exon 1 to exon 3 was performed by standard PCR (primers are marked with yellow arrowheads in Fig. S1a; the sequences are: P4: 5'-GGAGACTGACAGCAGGGGACAT-3', P5: 5'-GAGGAAGAGCACCACGTTAGCA-3'). The RT-PCR product was purified from agarose gels, and subsequently inserted into a plasmid using a TA cloning kit (pcDNA™3.1/V5-His TOPO® TA Expression Kit, Thermo, Rockford, IL, USA) according to the manufacturer's instruction. The inserted sequence was analyzed by a DNA analyzer (96-capillary 3730 xl DNA Analyzer, Thermo, Rockford, IL,USA).

Echocardiogram

Mice were anesthetized by 1 % isoflurane (Halocarbon, River Edge, NJ, USA) inhalation. Cardiac functions were measured in the presence or absence of isoproterenol (ISO, 10 mg/kg, 10 min) or atropine (10 mg/kg, 30 min) using the iE33 Echocardiography System (Philips Medical Systems, Bothell WA, USA) in M-mode. The parameters of fractional shortening (FS), ejection fraction (EF), end-diastolic diameter (EDD), end-systolic diameter (ESD), posterior wall (PW), inter ventricular septum (IVS) and heart rate (HR) were measured.

Subcellular Fractionation

Mouse hearts were harvested and left ventricles were dissected in ice-cold PBS. Ventricles were homogenized in the homogenizing buffer (20 mM Tris pH 7.4, 1 mM EDTA, 1x protease inhibitor complete (Roche)). For SR fraction, lysates were centrifuged at 12,000 g for 30 min to remove debris. Supernatants were diluted with the homogenizing buffer containing 300 mM KCl for 30 min on ice to dissolve myofibrillar proteins. After supernatants were centrifuged at 43,666 g for 30 min at 4 °C, pellets were resuspended in the lysis buffer (homogenizing buffer containing 250 mM sucrose and 10 mM histidine), and centrifuged again as described above. The resultant pellets were resolved in the lysis buffer, and stored at -20 °C (3). For the cytosolic fraction, lysates were centrifuged at 200,000 for 20 min at 4 °C. The Supernatant was stored at -20 °C. For the sarcolemmal fraction, lysates were centrifuged at 10,000 g for 10 min to remove the debris. Resultant supernatants were centrifuged at 200,000 g for 20 min at 4 °C. The pellets were resuspended in the homogenizing buffer containing 600 mM KCl to dissolve myofibrillar proteins. After the centrifugation at 200,000 g for 20 min at 4 °C for twice to remove the KCl buffer, the

resultant pellets were resolved in the lysis buffer and stored at -20 °C (23).

Calcium current recording

Isolated adult cardiomyocytes were incubated in the bath solution containing 145 mM tetraethylammonium chloride (TEA-Cl), 5 mM $CaCl_2$, 5 mM glucose, 10 mM HEPES, 1 mM $MgCl_2$, 5 mM CsCl, 1 mM 4-aminopyridine and 0.01 mM tetrodotoxin (TTX), pH 7.3 by TEA-OH, 300 mOsm. A patch electrode was filled with internal solution (130 mM CsCl, 5 mM $MgCl_2$, 10 mM EGTA, 20 mM HEPES, 3 mM Mg_2ATP and 0.3 mM Tris-GTP pH 7.3 by CsOH, 310 mOsm), and the electrode resistance was 1.5-2 MΩ. L-type calcium currents were elicited by a family of depolarizing steps from -60 mV to 40 mV for 200 ms at an increment of 10 mV per step. Current signals were recorded using an Axopatch 200B amplifier (Axon Instruments) with a filter set at 5 kHz via a digital-to-analog converter (Digidata 1322, Axon Instruments) controlled by pCLAMP 9.2 software.

Supplementary Table

Table S1. Echocardiographic measurements of WT (AC6$^{+/+}$), AC6 $^{\Delta N/\Delta N}$ and AC6$^{-/-}$ mice. Mice were first anesthetized by isoflurane (1 %) and then subjected to the measurements of end diastolic diameter (EDD), end systolic diameter (ESD), posterior wall (PW), interventricular septum (IVS), and left ventricular mass (LV)/body weight (BW). To determine the response to isoproterenol (ISO), mice were intraperitoneally injected with saline (Basal) or ISO (10 mg/kg) as indicated for 10 min, and then subjected to the measurements of heart rate, left ventricular ejection fraction (LVEF), and fraction shorting (FS). The data are expressed as the mean ± SEM. * P<0.05, ** P<0.01, compared to the corresponding wild-type group. $^{\#}$ P<0.05, compared to basal condition in each group. N indicates the number of mice in each group. AC6$^{+/+}$: N=11. AC6 $^{\Delta N/\Delta N}$: N=4. AC6$^{-/-}$: N=5

		**AC6$^{+/+}$**	**AC6 $^{\Delta N/\Delta N}$**	**AC6$^{-/-}$**
**EDD (cm)**		0.391 ± 0.008	0.405 ± 0.01	0.362 ± 0.009*
**ESD (cm)**		0.224 ± 0.005	0.258 ± 0.005**	0.204 ± 0.005*
**PW (cm)**		0.064 ± 0.002	0.065 ± 0.003	0.072 ± 0.01
**IVS (cm)**		0.085 ± 0.002	0.088 ± 0.005	0.084 ± 0.002
**LV/BW (%)**		0.375 0.009	0.379 0.022	0.371 0.016
**Heart rate (bpm)**	**Basal**	420.6 ± 4.1	422.2 ± 3.2	431.9 ± 2.9
	**ISO**	582.5 ± 9.3$^{\#}$	556.8 ± 9.3$^{\#}$	549.5 ± 11.0
**LVEF (%)**	**Basal**	80.2 ± 0.6	73.3 ± 1.5**	82.8 ± 1.9
	**ISO**	96.8 ± 0.4$^{\#}$	92.8 ± 0.8** $^{\#}$	92.4 ± 0.4** $^{\#}$

**FS (%)**	**Basal**	42.8 ± 0.6	35.0 ± 1.5**	43.8 ± 0.6
	**ISO**	69.9 ± 1.1#	60.0 ± 1.5** #	58.4 ± 0.7** #

Supplementary Figures

Figure S1

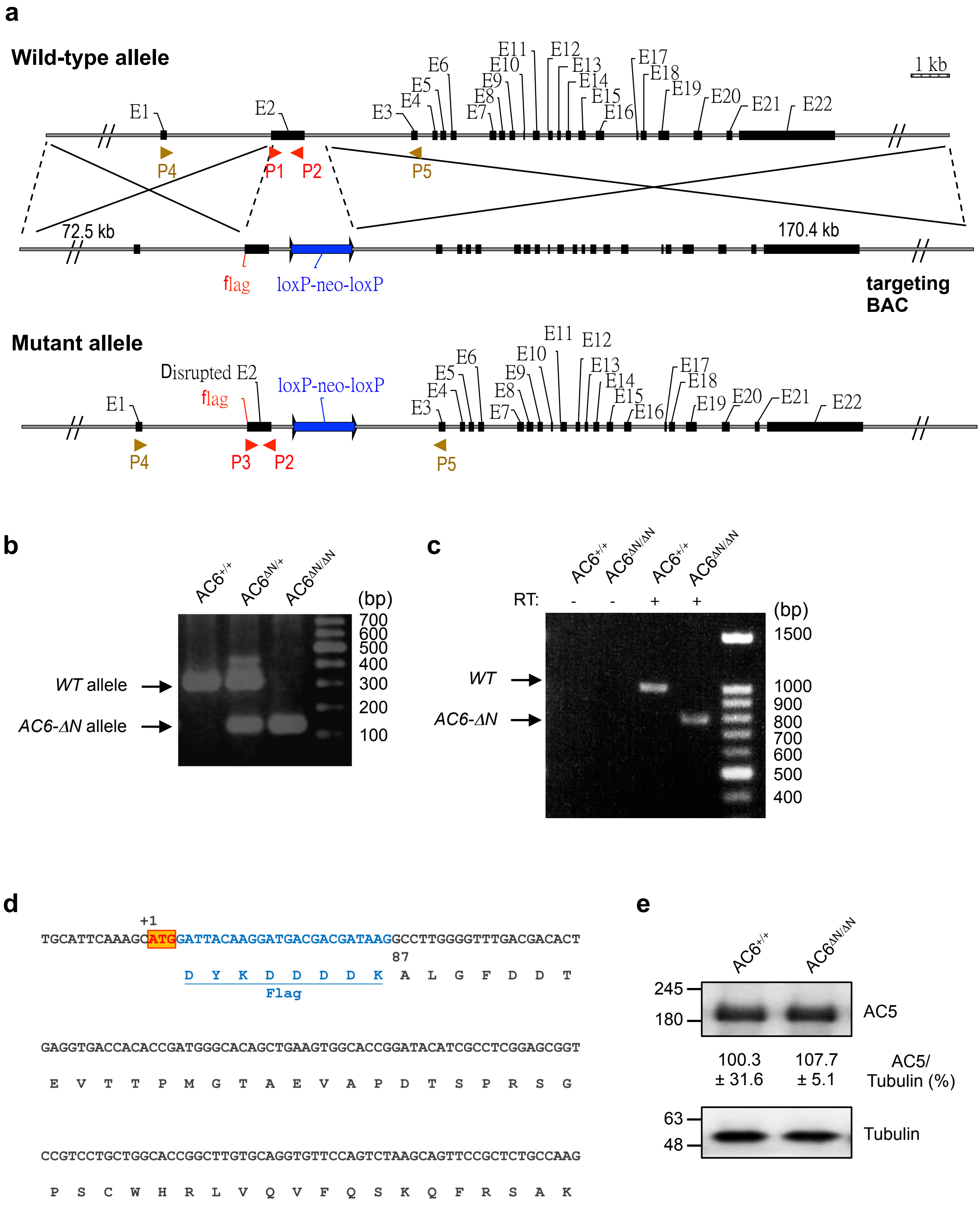

Figure S1. Targeted mutation of the mouse AC6 gene. a The mouse AC6 gene contains 22 exons. A map of the mutated BAC construct is shown. The DNA fragment encoding aa 2-86 was deleted from the BAC construct and replaced with a fragment encoding a flag tag. The arrowheads indicate the locations of genotyping primers. b A representative picture of the genotyping PCR products of $AC6^{+/+}$, $AC6^{\Delta N/+}$, and $AC6^{\Delta N/\Delta N}$. c A representative picture of RT-PCR analyses of transcripts collected from the heart of WT and $AC6^{\Delta N/\Delta N}$ mice using primers located in the exon 1 and the exon 3 of AC6. RT, reverse transcriptase. d Transcript from $AC6^{\Delta N/\Delta N}$ heart was amplified by RT-PCR using primers located in the exon 1 and the exon 3. The DNA sequence of the RT-PCR product is shown. The aa sequence is displayed beneath the DNA sequence. The translation start site is boxed. The flag-tag sequence is underlined. The aa sequence of AC6 starts at aa 87. e Expression of AC5 was analyzed using Western blot analysis. The level of AC5 in each group was normalized to that of tubulin (a loading control). The percentage of AC5 expression is normalized to the mean value in $AC6^{+/+}$ mice (N=3 per group). No significant difference between $AC6^{+/+}$ and $AC6^{\Delta N/\Delta N}$ mice was found. The data are presented as the mean ± SEM.

Figure S2

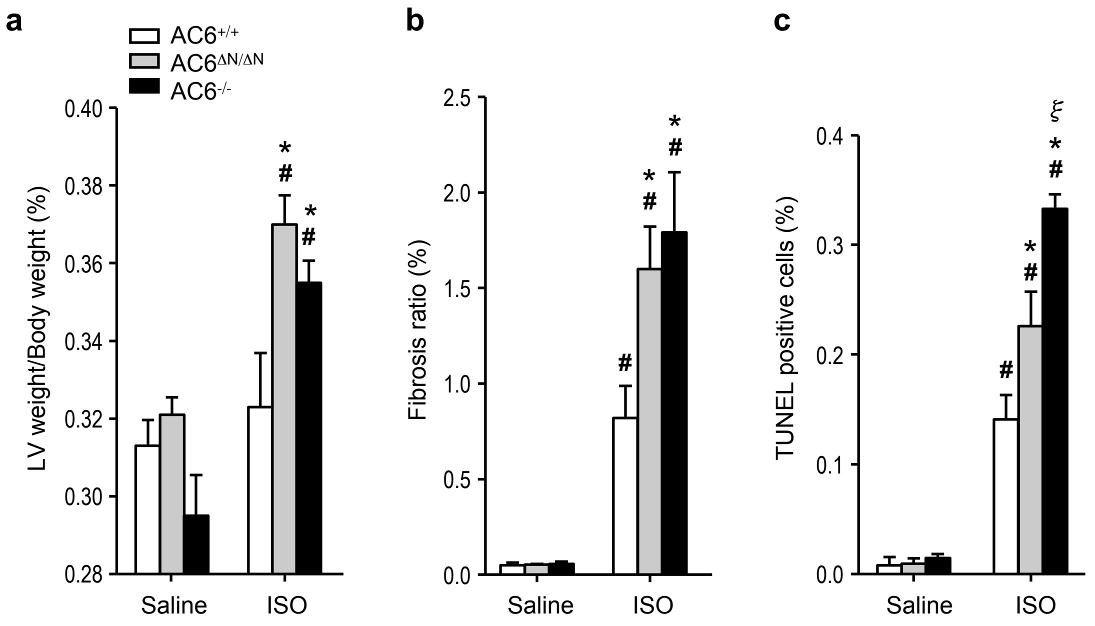

Figure S2. AC6$^{\Delta N/\Delta N}$ mice exhibited more cardiac hypertrophy, fibrosis, and apoptosis than WT mice in response to a single challenge of ISO injection. Mice were subjected to a single challenge of ISO (intraperitoneal injection, 10 mg/kg). a On day 8 after the ISO injection, the heart were carefully excised, weighed, and normalized by body weight. The left ventricle (LV) was removed and weighted. The data are expressed as the mean ± SEM (AC6$^{+/+}$/Saline, N=10; AC6$^{\Delta N/\Delta N}$/Saline, N=3; AC6$^{-/-}$/Saline, N=3; AC6$^{+/+}$/ISO, N=5; AC6$^{\Delta N/\Delta N}$/ISO, N=4; AC6$^{-/-}$/ISO, N=5). * P<0.05 vs. AC6$^{+/+}$ in the indicated condition. $^{\#}$ P<0.05 vs. the basal level in each group. b The Sirus red-positive region was quantified as described in the Methods section. The data are expressed as the mean ± SEM (AC6$^{+/+}$/Saline, N=3; AC6$^{\Delta N/\Delta N}$/Saline, N=3; AC6$^{-/-}$/Saline, N=3; AC6$^{+/+}$/ISO, N=5; AC6$^{\Delta N/\Delta N}$/ISO, N=6; AC6$^{-/-}$/ISO, N=3). * P<0.05 vs. AC6$^{+/+}$. $^{\#}$ P<0.05 vs. the basal level. c The TUNEL assay was conducted. The TUNEL- positive cells were quantified as described in the Methods section. The data are expressed as the mean ± SEM (AC6$^{+/+}$/Saline, N=3; AC6$^{\Delta N/\Delta N}$/Saline N=3; AC6$^{-/-}$/Saline, N=3; AC6$^{+/+}$/ISO, N=3; AC6$^{\Delta N/\Delta N}$/ISO N=4; AC6$^{-/-}$/ISO, N=4). * P<0.05 vs. AC6$^{+/+}$. $^{\#}$ P<0.05 vs. the basal level. $^{\xi}$ P<0.05 vs. AC6$^{\Delta N/\Delta N}$.

Figure S3

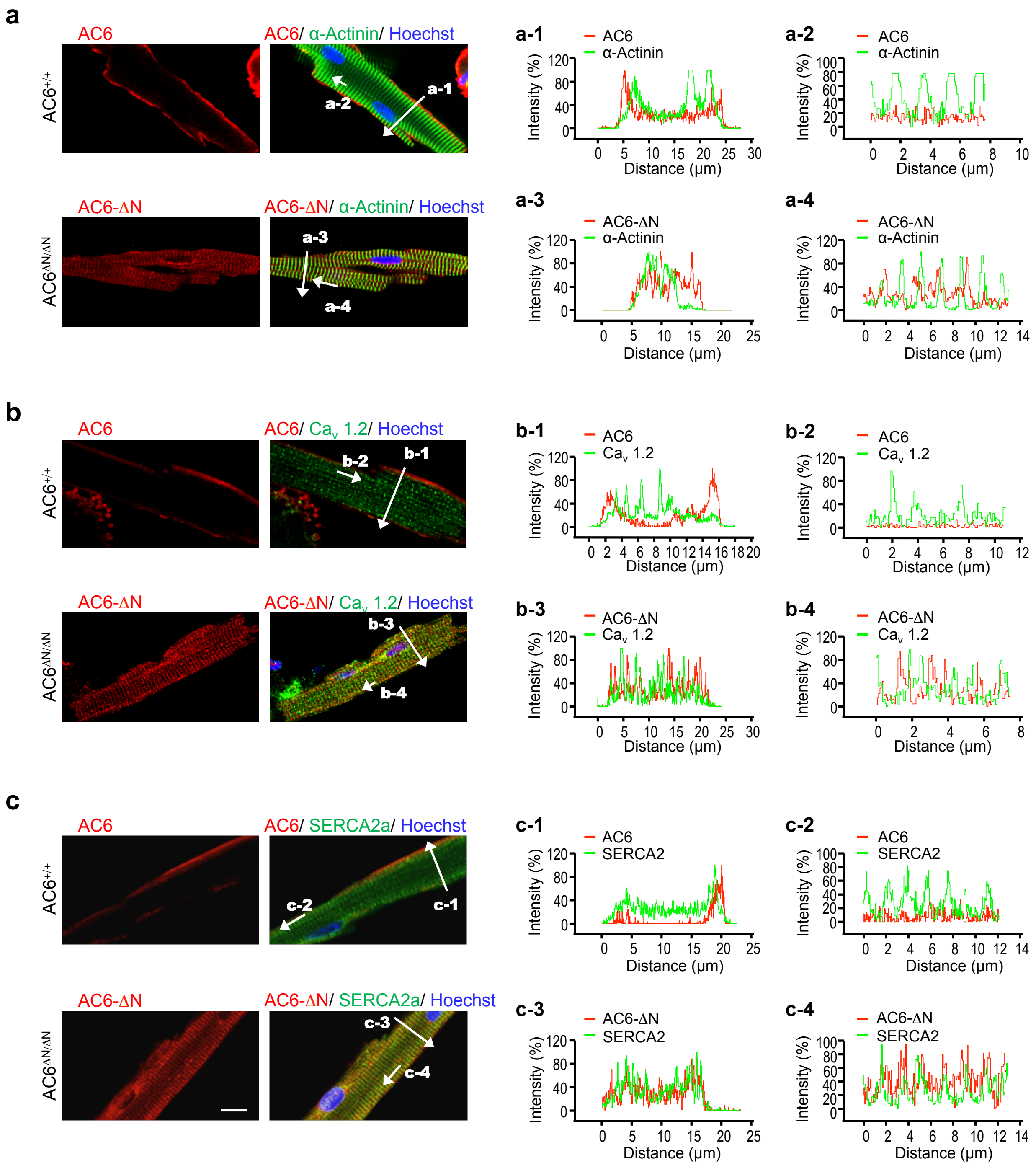

a
AC6
AC6/ α-Actinin/ Hoechst
AC6+/+
a-1
a-2
AC6-ΔN
AC6-ΔN/ α-Actinin/ Hoechst
AC6ΔN/ΔN
a-3
a-4
AC6
α-Actinin
AC6-ΔN
Intensity (%)
Distance (μm)
b
AC6/ Cav 1.2/ Hoechst
AC6-ΔN/ Cav 1.2/ Hoechst
b-1
b-2
b-3
b-4
Cav 1.2
c
AC6/ SERCA2a/ Hoechst
AC6-ΔN/ SERCA2a/ Hoechst
c-1
c-2
c-3
c-4
SERCA2

Figure S3. Cellular localization of AC6 and flag-AC6-ΔN in cardiomyocytes. Localization of AC6 (red) or flag-AC6-ΔN (red), and α-actinin (a, green), $Ca_v1.2$ (b, green), and SERCA2a (c, green) in adult cardiomyocytes was assessed by immunofluorescence staining as shown in Figure 3. Scale bar, 10 μm. Image analyses (a-1 - 4, b-1 - 4, c-1 - 4) are presented in the rightmost panels, which show the merged signals of AC6 (red curves), flag- AC6-ΔN (red curves), α-actinin (green curves), $Ca_v1.2$ (green curves), and SERCA2a (green curves). White arrows in the merged images indicate the sampling areas of the corresponding image.

Figure S4

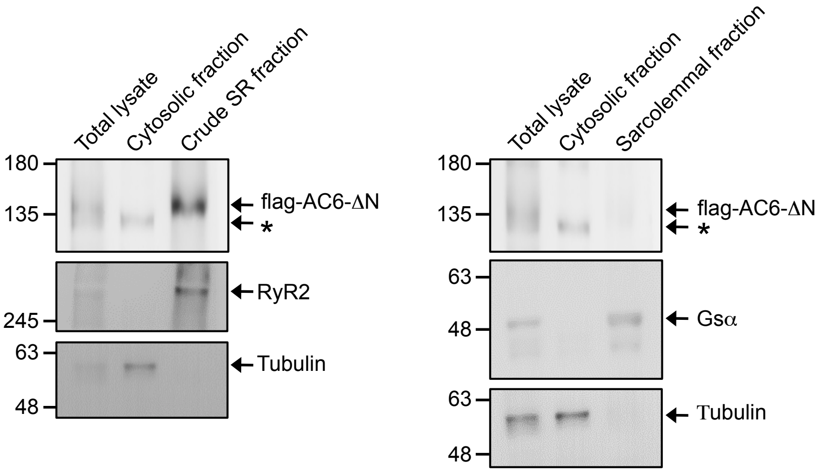

Figure S4. AC6-ΔN distribution detected by biochemical fractions of heart lysate. Tissue lysate was prepared from AC6$^{\Delta N/+}$ heterozygous mice. The total lysate, cytosolic fraction, sarcolemmal membrane fraction, and crude sarcoplasmic reticulum (SR) fraction were prepared as described in the section of Methods and Materials. Equal amount (10 µg/lane) of the indicated samples were analyzed by SDS-PAGE/ Western blot analysis. Tubulin, Gsα, and RyR2 are markers for the cytosolic fraction, the sarcolemmal membrane, and the crude SR fraction, respectively. The AC6-ΔN signal detected by anti-flag antibody was enriched in the crude SR fraction. * indicates the non-specific signals from the anti-flag antibody.

Figure S5

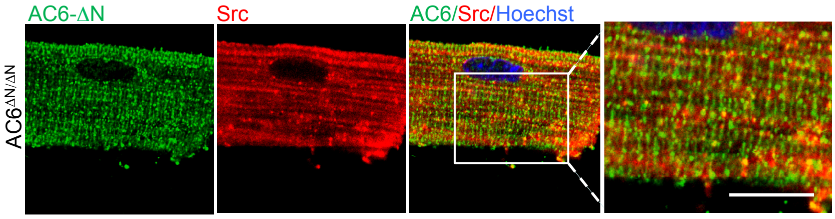

Figure S5. Cellular localization of flag-AC6-ΔN and Src in cardiomyocytes. Adult AC6$^{ΔN/ΔN}$ cardiomyocytes were immunostained for flag-AC6-ΔN (green) and Src (red). Scale bar, 10 µm. The rightmost panel shows the enlarged, merged image of the field in the white rectangle. Scale bar, 10 µm.

Figure S6

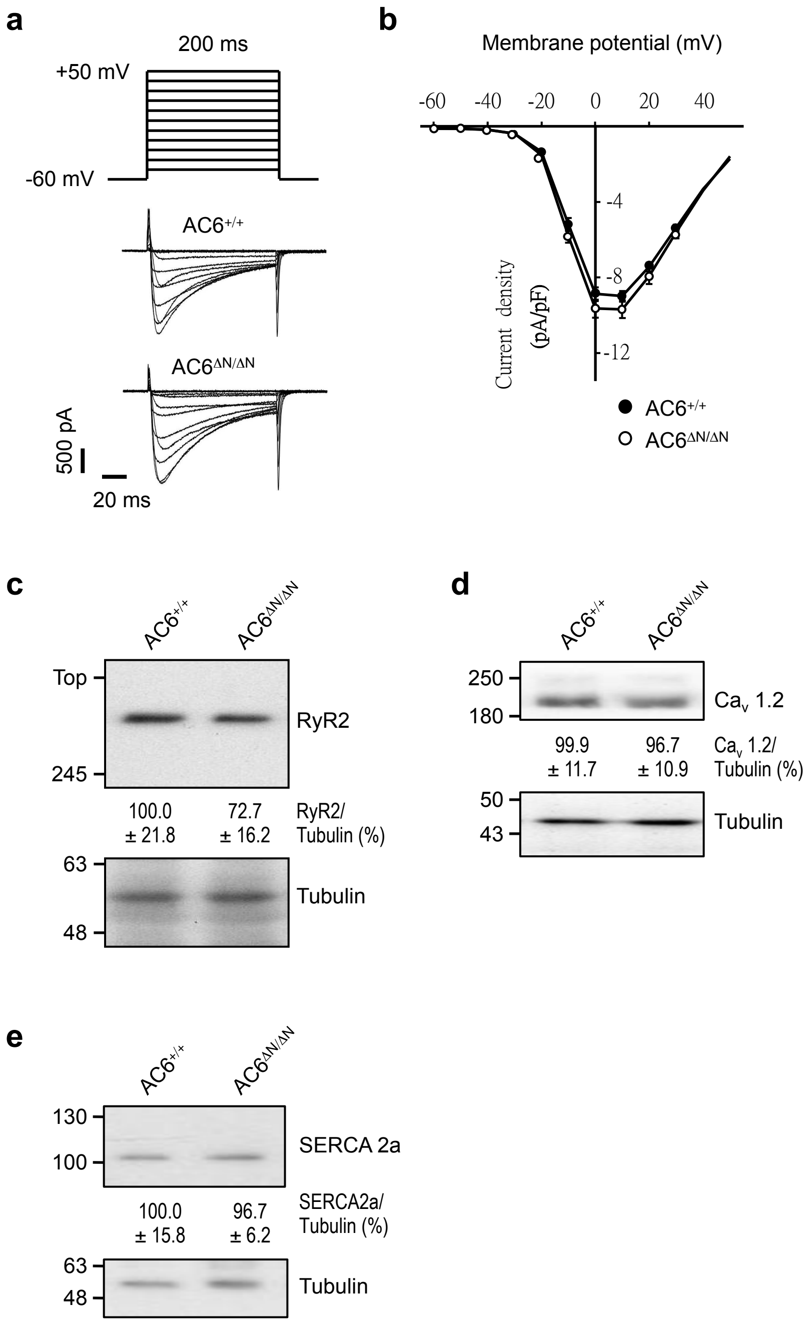
a
200 ms
+50 mV
-60 mV
AC6+/+
AC6ΔN/ΔN
500 pA
20 ms
b
Membrane potential (mV)
-60 -40 -20 0 20 40
-4
-8
-12
Current density
(pA/pF)
AC6+/+
AC6ΔN/ΔN
c
AC6+/+
AC6ΔN/ΔN
Top
245
RyR2
100.0
± 21.8
72.7
± 16.2
RyR2/
Tubulin (%)
63
48
Tubulin
d
AC6+/+
AC6ΔN/ΔN
250
180
Cav 1.2
99.9
± 11.7
96.7
± 10.9
Cav 1.2/
Tubulin (%)
50
43
Tubulin
e
AC6+/+
AC6ΔN/ΔN
130
100
SERCA 2a
100.0
± 15.8
96.7
± 6.2
SERCA2a/
Tubulin (%)
63
48
Tubulin

Figure S6. Levels of the L-type Ca^{2+} current and the Ca^{2+} handling proteins in $AC6^{\Delta N/\Delta N}$ cardiomyocytes. Adult cardiomyocytes were prepared from the indicated mice. a, b L-type calcium currents were elicited by a series of depolarizing steps from -60 mV to 40 mV for 200 ms in increments of 10 mV in the presence of tetrodotoxin (TTX), a sodium channel blocker. The voltage-current relationship curves of L-type Ca^{2+} currents were plotted in b. The L-type calcium channel-evoked Ca^{2+} current was similar between $AC6^{+/+}$ and $AC6^{\Delta N/\Delta N}$ cardiomyocytes (N=3). c-e Total lysates were prepared from the hearts of the indicated mice (2 -5 months old), and subjected to Western blot analysis to examine the amount and the level of the indicated protein. Tubulin was used as a loading control. The amounts of the indicated proteins were normalized to the amount of the corresponding loading control. The percentage of expression is normalized to the mean expression in $AC6^{+/+}$ mice (c, N=6; d, N=3; e, N=6 per group) and is presented beneath the corresponding Western blot. The data are expressed as the mean ± SEM. * $P<0.05$ vs. $AC6^{+/+}$.
